# Validation of new transmission detector transmission factors for online dosimetry: an experimental study

**DOI:** 10.1186/s13014-018-1106-y

**Published:** 2018-08-24

**Authors:** So-Yeon Park, Jong Min Park, Jung-in Kim, Sungyoung Lee, Chang Heon Choi

**Affiliations:** 1 Department of Radiation Oncology, Veterans Health Service Medical Center, Seoul, Republic of Korea; 2 Institute of Radiation Medicine, Seoul National University Medical Research Center, Seoul, Republic of Korea; 3 Department of Radiation Oncology, Seoul National University Hospital, Seoul, Republic of Korea; 4 Biomedical Research Institute, Seoul National University Hospital, Seoul, Republic of Korea

**Keywords:** Transmission detector, Transmission factors, Portal dosimetry, Online dosimetry

## Abstract

**Background:**

The demand for dose verification during treatment has risen with the increasing use of intensity-modulated radiation therapy (IMRT) and volumetric modulated arc therapy (VMAT) in modern radiation therapy. This study aims to validate the transmission factors of a new transmission detector, the Dolphin online monitoring system (IBA Dosimetry, Schwarzenbruck, Germany), for clinical use.

**Methods:**

The transmission factors of the Dolphin detector were evaluated using 6 MV, 6 flattening filter free (FFF), 10 MV, and 10 FFF clinical beams from a TrueBeam STx linear accelerator system. Two-dimensional (2D) dose distributions were measured through portal dosimetry with and without Dolphin to derive the transmission factors. The measurements were performed using 10 IMRT and 10 VMAT treatment plans. The transmission factors were calculated using a non-negative least squares problem solver for the 2D dose matrix. Normalized plans were generated using the derived transmission factors. Patient-specific quality assurance with normalized plans was performed using portal dosimetry and an ArcCheck detector to verify the transmission factors. The gamma passing rates were calculated for the 2%/2 mm and 1%/1 mm criteria.

**Results:**

The transmission factors for the 6 MV, 6 FFF, 10 MV, and 10 FFF beams, were 0.878, 0.824, 0.913, and 0.883, respectively. The average dose difference between the original plan without Dolphin and the normalized plan with Dolphin was less than 1.8% for all measurements. The mean passing rates of the gamma evaluation were 98.1 ± 2.1 and 82.9 ± 12.6 for the 2%/2 mm and 1%/1 mm criteria, respectively, for portal dosimetry of the original plan. In the case of the portal dosimetry of the normalized plan, the mean passing rates of the gamma evaluation were 97.2 ± 2.8 and 79.1 ± 14.8 for the 2%/2 mm and 1%/1 mm criteria, respectively.

**Conclusions:**

The Dolphin detector can be used for online dosimetry when valid transmission factors are applied to the clinical plan.

## Background

In modern radiation therapy, intensity-modulated radiation therapy (IMRT), volumetric modulated arc therapy (VMAT), and stereotactic ablative radiotherapy (SABR) are used for precise radiation delivery [1, 2]. These techniques can generate a sophisticated dose distribution, delivering high doses to the target with lower doses to critical organs. Such dose distributions are delivered using a beam sequence with various degrees of freedom to address an increasingly conformal dose distribution [3]. Therefore, they require a comprehensive quality assurance (QA) testing to verify the dose delivery [4–6]. These QA methods can detect possible errors, which can cause serious side effects for patients [7, 8]. Hence, pretreatment patient-specific dosimetry is essential [9].

In recent decades, IMRT verification techniques have been developed to increase the efficiency and accuracy of associated QA procedures [10]. Plan verification is generally performed using detector array systems, ion chamber, or film before first treatment. However, such pretreatment plan verifications have certain limitations [11, 12]. Verification is performed only once without the patient and before the first fraction on the assumption that the dose is delivered with no change or error over the course of treatment. However, this approach does not ensure that the planned dose is delivered to the patient during all treatment fractions because the mechanical conditions of the treatment machine can vary.

In modern radiotherapy, the focus has shifted toward adaptive radiotherapy, and an increasing demand for online dose verification of dose delivery has been observed [13]. Dose-distribution prediction by analyzing the machine delivery log file has been introduced for online dose verification [14]. Reconstructed three-dimensional (3D) dose verification models have also been developed for the same purpose [15–17]. In other ways, the 3D dose has been reconstructed from a computed tomography (CT) dataset based on measurement data and 3D dose calculation software [18].

Accordingly, transmission detectors have been developed to address these demands, and several commercial products are now available for clinical purposes (e.g., IQM (iRT Systems GmbH, Koblenz, Germany), OCTAVIUS III DAVID (PTW Freiburg GmbH, Freiburg, Germany), Delta4 Discover (ScandiDos AB, Uppsala, Sweden), and the Dolphin online monitoring system (IBA Dosimetry, Schwarzenbruck, Germany) [11, 19, 20].

Dolphin is a monitoring system with transmission detectors. The measurement data can be reconstructed to a 3D dose distribution on a 3D-CT image set using COMPASS software (IBA Dosimetry, Schwarzenbruck, Germany) [21, 22]. Dolphin can be used for pretreatment QA as well as during each fraction. However, Dolphin has some shortcomings, such as beam hardening scatter and electron contamination [11]. Dolphin has a technical potential function for online dosimetry, which can be performed by mounting a detector on the gantry during treatment [15]; however, it is not allowed for Dolphin. Note that in treatment planning, the dose is calculated without considering the attenuation by the detector. The transmission detector is located between the beam source and the patient [12], and the radiation beam is perturbed by the material and thickness of the transmission detector [23]. Until now, the treatment planning system (TPS) cannot address the beam attenuation factor of the transmission detector when a transmission detector is installed for online dosimetry. In other words, TPS does not support plan optimization and dose calculation for Dolphin. Therefore, the use of Dolphin in actual treatments is restricted [9].

Cheung et al. recently presented the square field transmission factors for 6 MV, 6 flattening filter free (FFF), 10 MV, and 10 FFF clinical beams. These factors were previously available only for the static field used in 3D conformal radiotherapy. However, online dosimetry is more important for IMRT and VMAT [11]. The dosimetric leaf gap (DLG) and the multileaf collimator (MLC) transmission are important parameters for the optimization and dose calculation of intensity-modulated fields delivered by the MLC [24].

This study derived transmission factors of the Dolphin online monitoring system for IMRT and VMAT plan using 6 MV, 6 FFF, 10 MV, and 10 FFF clinical beams. We generated a normalized plan, which was normalized by transmission factors. To verify the transmission factors, the gamma index of the normalized plan was evaluated using two independent QA devices. In addition, the DLG and MLC transmission factors were individually measured to confirm the change of their characteristics by the Dolphin system for each available energy.

## Methods

### Transmission detector

The Dolphin transmission detector was a 2D array with 1513 plane-parallel ionization chambers. The volume of each chamber was 0.016 cm^3^ (diameter: 3.2 mm, height: 2 mm). The chambers were arranged with a 5 mm spacing in the central area (i.e., within the inner 15 × 15 cm^2^ region) and with a 10 mm spacing in the outer detector area (i.e., corresponding size in the isocenter plane: chamber diameter: 4.89 mm, 7.63 mm, and 15.26 mm spacing in the central and outer area, respectively).

The physical size of the detector was 24.3 × 24.3 cm^2^. A field size of up to 40 × 40 cm^2^ can be measured by installing the interface mount on the gantry head (at 65.5 cm from the source for TrueBeam STx system (Ver. 2.5, Varian Medical Systems, Inc., Palo Alto, CA)). The device incorporates a copper buildup plate with 1.5 mm thickness [11].

### Measurement setup and energies

**Fig. 1:**
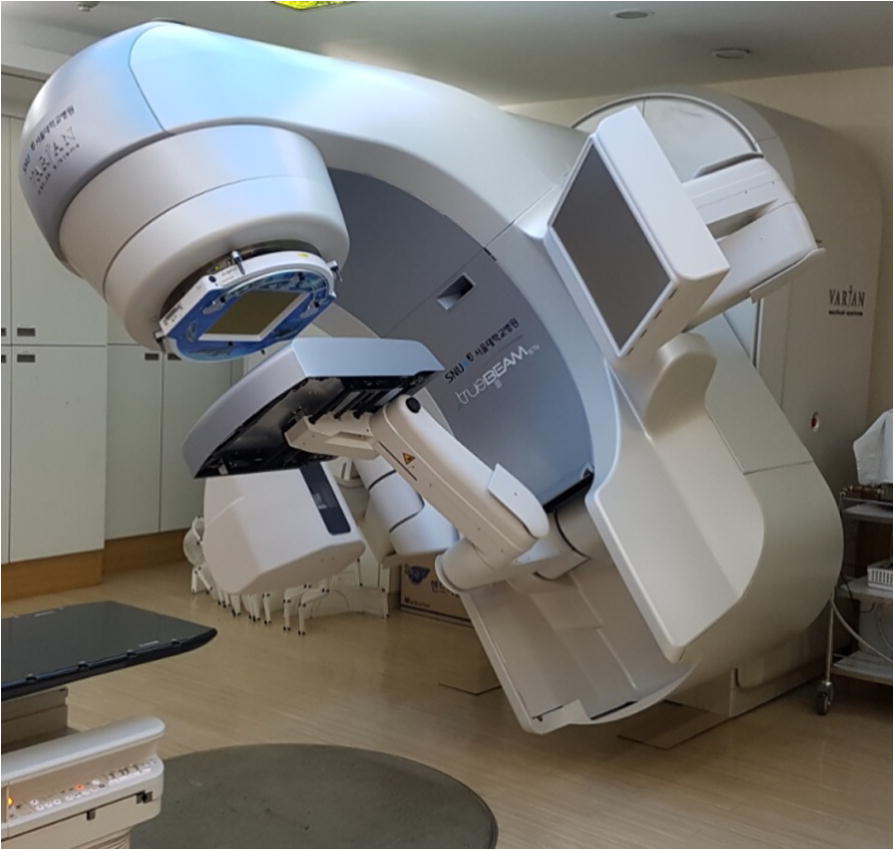
Measurement device and linear accelerator: transmission detector and TrueBeam STx

Figure 1 shows the basic measurement setup involving the Dolphin detector and a linear accelerator. All measurements were conducted using the TrueBeam STx system equipped with a high-definition 120-leaf multileaf collimator. The four energies (i.e., 6 MV, 6 FFF, 10 MV, and 10 FFF) were used for the transmission factor measurement. The COMPASS software was not commissioned for 15 MV; therefore, 15 MV was excluded from the transmission factor measurement. Table 1 lists the values of the depth of maximum dose (***D***_**max**_), the percentage depth dose at 10 cm depth (***PDD***_**10**_), and tissue phantom ratio at the depths of 20 and 10 cm (***TPR*** **20/10**) with and with Dolphin for the four examined energies.

**Table 1:** Depth of maximum dose (*D*_max_) and percentage depth dose at 10 cm depth (*PDD*_10_)

Energy	^a^*D*_max_ (cm)		^b^*PDD*_10_ (%)		^d^*TPR* 20/10	
	With Dolphin	Without Dolphin	With Dolphin	Without Dolphin	With Dolphin	Without Dolphin
6 MV	1.5	1.5	66.2	66.1	0.668	0.667
6 ^c^FFF	1.3	1.3	63.2	63.0	0.633	0.632
10 MV	2.1	2.1	73.6	73.5	0.739	0.737
10 FFF	2.3	2.3	70.9	70.6	0.694	0.692

^a^*D*_*max*_ depth of maximum dose, ^b^*PDD*_10_ percentage depth dose at 10 cm depth, ^c^*FFF* flattening filter free, and ^d^*TPR* 20/10 tissue phantom ratio at the depths of 20 and 10 cm

### Transmission factor and output factor measurements for square fields

The transmission factors of regular square fields were measured to evaluate the field size dependency. The transmission factor measurement was performed with 0.125-cc ionization chambers (TN31010, PTW, Freiburg, Germany) and an electrometer (Uniods E, PTW, Freiburg, Germany) in a solid water phantom (SWP, Virtual Watert™, Radiation Products Design, Inc., Albertville, MN) for various square fields (i.e., 3 × 3, 5 × 5, 7 × 7, 9 × 9, 10 × 10, 12 × 12, 15 × 15, and 20 × 20 cm^2^) and four energies. The SWP measured 30 × 30 cm^2^. The backscatter material behind the chamber was 5 cm thick of the SWP. The ionization chambers were located at the isocenter with a 5 cm depth for all energies. The output factors were also measured for eight square fields and four energies with and without Dolphin. The output factors were normalized to a 10 × 10 cm^2^ field size.

### Dosimetric leaf gap and multileaf collimator transmission factor

The DLG and the transmission factor of the MLC were examined to verify the IMRT and VMAT plans with and without Dolphin. The DLG was calculated by extrapolating the measurements of sweeping gap fields with varying widths (i.e., 2, 4, 6, 10, 14, 16, and 20 mm) [25]. The MLC transmission ratio was defined as the ratio of the dose in the MLC leaves to the open field dose, which was calculated with and without Dolphin.

### Dolphin transmission factors using IMRT and VMAT plans

Subsequently, 10 IMRT and 10 VMAT plans were randomly selected for the four energies to calculate the transmission factors of the clinical beams. Each IMRT plan included five fields. VMAT plans with full and partial arcs were selected. IMRT and VMAT plans for various treatment sites (i.e., brain, head and neck, lung, spine, abdomen, and pelvic region) were chosen with delivery doses of 1.8–17 Gy. Four of the VMAT plans were generated for SABR. The jaw size was approximately 9 × 9 cm^2^ to 25 × 25 cm^2^ for both IMRT and VMAT plans. For SABR, the jaw size was approximately 3 × 3 cm^2^ to 6 × 6 cm^2^. A jaw tracking option was not applied.

All the IMRT and VMAT plans were generated by Eclipse 13.7 (Varian Medical Systems, Inc., Palo Alto, CA) based on the Acuros XB algorithm. A patient-specific verification plan was created based on the clinical patient plan to use the portal dosimetry system. Verification plans have a predicted fluence map on the electronic portal imaging device (EPID). The predicted fluence map can be compared with the measured fluence map by the EPID. The portal dose image prediction (PDIP, Ver 13.7, Varian Medical Systems, Inc., Palo Alto, CA) algorithm was used for portal dosimetry.

The transmission factors of the clinical beams were measured via portal dosimetry and an ArcCheck detector (Sun Nuclear Corporation (SNC), Melbourne, FL) using SNC patient software (Ver. 6.6, SNC, Melbourne, FL). An aS1200 EPID was used for portal dosimetry. The active area of the EPID was 40 × 40 cm^2^ for portal dosimetry, with a 0.336 mm resolution 2D pixel array (1190 × 1190) [26]. The EPID was placed in the isocenter plane for measurement. Portal dosimetry was performed for the 25 × 25 cm^2^ field size with and without Dolphin to investigate the perturbation of measurement by the interior design of Dolphin. The uniformity was compared to two square dose distributions of EPID. The uniformity was defined as the average difference dose distribution relatively with and without Dolphin for pixel by pixel in 80% area of the field size.

### Transmission factor calculation and verification for IMRT and VMAT

**Fig. 2:**
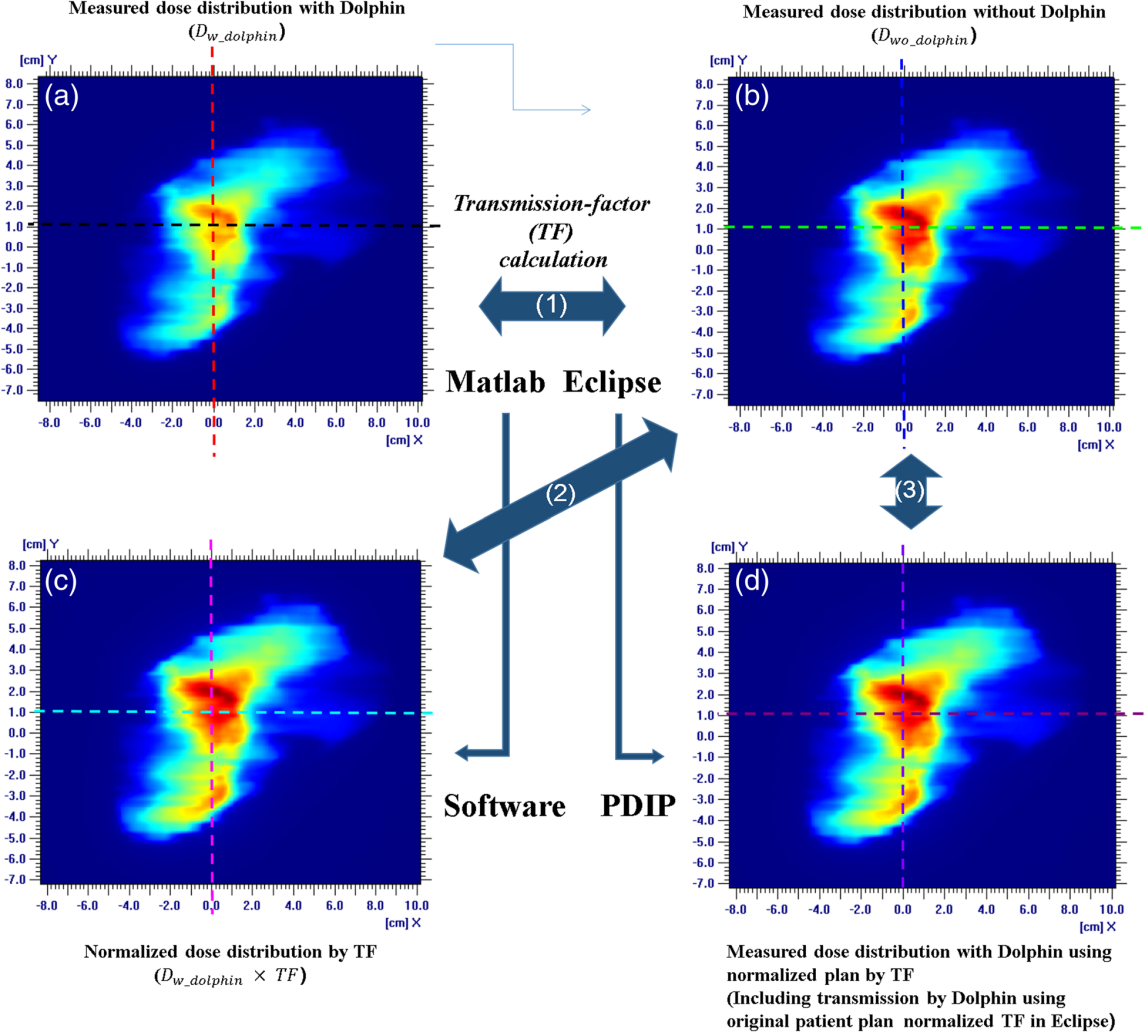
Dose distribution: **a**) measurement of portal dosimetry with Dolphin; **b**) measurement of portal dosimetry; **c**) dose distribution, where the measurement of portal dosimetry with Dolphin is normalized by the transmission factor (TF); and **d**) measurement of portal dosimetry for the normalized plan with Dolphin (measurement value: calibration unit (CU))

The transmission factors were calculated as follows using the non-negative least-squares (NNLS) problem solver (Fig. 2(1)) [27]:

1 $$ \mathit{\arg}{\mathit{\min}}_{TF}\ {\left\Vert {D}_{wo\_ dolphin}\times TF-{D}_{w\_ dolphin}\right\Vert}_2\kern0.5em \mathrm{subject}\kern0.5em \mathrm{to}\kern0.5em TF\kern0.5em \ge \kern0.5em 0, $$

where, *D*_*wo* _ *dolphin*_ (Fig. 2b) is the dose distribution (2D matrix) measured without the Dolphin detector; *TF* is the transmission factor defined as the ratio of the transmitted radiation intensity to the incident radiation intensity; *D*_*w* _ *dolphin*_ (Fig. 2a) is the dose distribution measured with the Dolphin; and ‖·‖_2_ denotes the Euclidean norm. The transmission factors were calculated for each field for all plans. Furthermore, the calculated transmission factors of all fields were averaged for each energy. The dose difference between and *D*_*w* _ *dolphin*_ and *D*_*wo* _ *dolphin*_ × *TF* (Fig. 2c) was calculated for each dose distribution to verify the transmission factors. The in-house program developed using MATLAB (R2015b, MathWorks, USA) was used for the NNLS calculation to obtain the average of the transmission factors and the dose difference between the dose matrices (Fig. 2. (2)).

The transmission factors were verified by measurement. For verification, normalized plans were generated by normalizing the original plan. This normalization was performed using the changing normalization value of the plan normalization mode in Eclipse. The normalization values were obtained by multiplying the original normalization value by the transmission factor of each energy (Fig. 2 (3)). After normalization, the monitoring unit (MU) of the normalized plans was equal to the MU of the original plans multiplying normalization values.

A patient-specific QA of the normalized plans was performed using portal dosimetry and an ArcCheck detector. The dose difference and the gamma index were evaluated. The dose difference between the two plans was calculated, excluding the below-10% dose. Two criteria were applied for the gamma analysis: 2% dose difference (DD) and 2 mm distance to agreement (DTA) and 1% DD and 1 mm DTA. The threshold was set to 10% dose.

The gamma values were calculated to compare the QA results for the original plan and the normalized plan with the Dolphin detector for both QA devices (e.g., portal dosimetry and ArcCheck). The gamma index evaluation was performed for the predicted dose of the original plan and the measurement for the normalized plan with the Dolphin detector.

## Results

### Transmission factor and output factor measurements for square fields

Table 2 lists the measured Dolphin transmission factors for various square fields and each beam energy. The measured transmission factors for a 10 × 10 cm^2^ field size were 0.893, 0.883, 0.914, and 0.906 for the 6 MV, 6 FFF, 10 MV, and 10 FFF cases, respectively. For the square field, the measured transmission factors decreased with the increasing field size. Table 3 lists the output factors for various square fields and each beam energy with and without Dolphin. The relative photon outputs without Dolphin exhibited a larger variation compared to those with Dolphin. The output factors had a larger range with a decreasing energy.

**Table 2:** Measured Dolphin transmission factors for the square fields and each beam energy

Field size (cm^2^)	Beam energy			
	6 MV	6 ^a^FFF	10 MV	10 FFF
3 × 3	0.946	0.935	0.948	0.941
5 × 5	0.932	0.921	0.936	0.928
7 × 7	0.916	0.904	0.925	0.917
9 × 9	0.901	0.890	0.920	0.911
10 × 10	0.893	0.883	0.914	0.906
12 × 12	0.887	0.873	0.909	0.902
15 × 15	0.877	0.863	0.903	0.896
20 × 20	0.866	0.854	0.897	0.891

^a^*FFF* flattening filter free

**Table 3:** Output factors for the square fields and each beam energy with and without Dolphin

Field size (cm^2^)	Beam energy							
	Output factor with Dolphin				Output factor without Dolphin			
	6 MV	6 ^a^FFF	10 MV	10 FFF	6 MV	6 FFF	10 MV	10 FFF
3 × 3	0.882	0.897	0.881	0.919	0.833	0.847	0.849	0.886
5 × 5	0.930	0.941	0.936	0.963	0.892	0.902	0.914	0.940
7 × 7	0.966	0.971	0.968	0.981	0.942	0.949	0.956	0.970
9 × 9	0.991	0.991	0.991	0.995	0.982	0.984	0.984	0.990
10 × 10	1.000	1.000	1.000	1.000	1.000	1.000	1.000	1.000
12 × 12	1.019	1.012	1.016	1.008	1.026	1.024	1.021	1.013
15 × 15	1.039	1.028	1.034	1.017	1.059	1.051	1.046	1.029
20 × 20	1.068	1.048	1.057	1.029	1.102	1.084	1.077	1.047

^a^*FFF* flattening filter free

### Dolphin transmission factors using IMRT and VMAT plans

Table 4 presents the MLC transmission factor and the DLG for each beam energy with and without Dolphin. The difference in the MLC transmission factors with and without Dolphin was within 1% for all energies. The DLG factors were almost identical with and without Dolphin. The maximum difference was 0.05 mm, which was obtained for 6 MV.

**Table 4:** MLC transmission factors and DLG for each beam energy with and without Dolphin

Energy	^a^MLC transmission factor	^b^DLG (mm)	MLC transmission factor	DLG (mm)
	With Dolphin		Without Dolphin	
6 MV	0.017	0.42	0.011	0.37
6 ^c^FFF	0.014	0.28	0.006	0.24
10 MV	0.022	0.45	0.013	0.5
10 FFF	0.019	0.38	0.009	0.35

^a^*MLC* multileaf collimator, ^b^*DLG* dosimetric leaf gap, and ^c^*FFF* flattening filter free

### Dolphin transmission factors using IMRT and VMAT plans

**Fig. 3:**
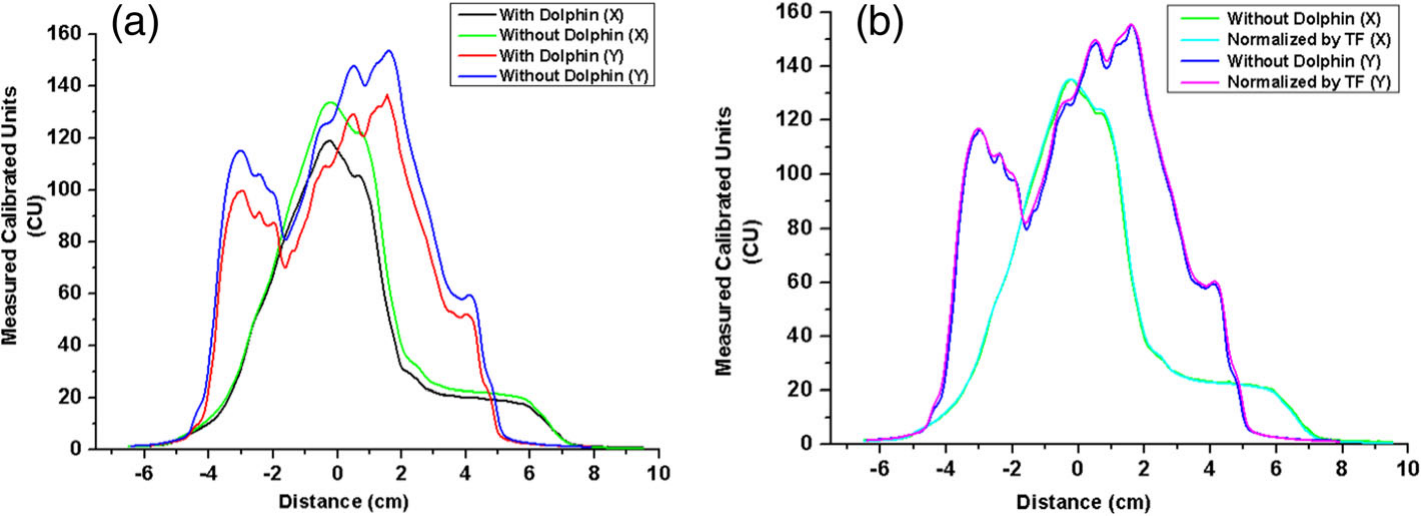
Dose profile: **a**) measured dose profile of the original plan with and without Dolphin (X-direction with Dolphin: black line, X-direction without Dolphin: red line, Y-direction with Dolphin: green line, and Y-direction without Dolphin: blue line) and **b**) dose distribution normalized by the transmission factor (TF) without Dolphin measurement (X-direction without Dolphin: green line, X-direction of the normalized dose: cyan line, Y-direction without Dolphin: blue line, and Y-direction of normalized dose: magenta line) (measurement value: calibration unit (CU))

Figure 2a and b show the measured dose distribution obtained via portal dosimetry with and without Dolphin, respectively, to represent the VMAT case. Figure 3a depicts the profile of each dose distribution. As a relative comparison, the dose distribution and the profile were almost identical. However, in absolute comparison, they exhibited remarkable differences. Table 5 lists the transmission factors for each energy and technique, which are the averages of the transmission factors calculated for each plan using Eq. (1). The differences between the IMRT and VMAT transmission factors were negligible. The average values were 0.878, 0.825, 0.913, and 0.883 for the 6 MV, 6 FFF, 10 MV, and 10 FFF cases, respectively.

**Table 5:** Calculated transmission factors for IMRT, VMAT, and SABR

Energy	^a^IMRT	^b^VMAT	^c^SABR
6 MV	0.878 ± 0.002	0.877 ± 0.002	0.878 ± 0.006
6 ^d^FFF	0.825 ± 0.003	0.823 ± 0.001	0.827 ± 0.007
10 MV	0.912 ± 0.003	0.914 ± 0.001	0.913 ± 0.009
10 FFF	0.883 ± 0.002	0.882 ± 0.003	0.885 ± 0.005

^a^*IMRT* intensity-modulated radiation therapy, ^b^*VMAT* volumetric modulated arc therapy, ^c^*SABR* stereotactic ablative radiotherapy, and ^d^*FFF* flattening filter free

The standard deviation was in the range of 0.1–0.3%. The maximum variation between the calculated transmission factors was less than 0.8% for all energies for VMAT and IMRT.

Figure 2c shows the dose distribution obtained with normalization by the transmission factor. Figure 3b illustrates the dose profile comparison between the original and normalized plans. The average dose difference between measurement and expectation was below 0.8%, whereas the maximum was 1.3%.

The uniformity of the 25 × 25 cm^2^ field was 0.8% with and without Dolphin. The dose distributions were approximately identical.

### Transmission factor verification

**Fig. 4:**
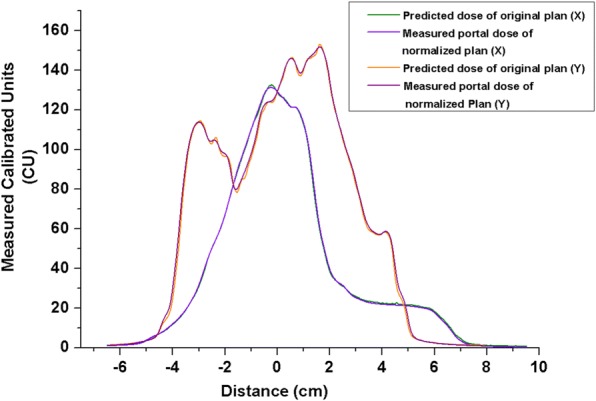
Dose profile for the predicted dose of the original plan and the measurement dose of the normalized plan with Dolphin for verification: X-direction of the predicted dose of original plan: dark green line; X-direction of the measurement dose of the normalized plan with Dolphin: violet line; Y-direction of the predicted dose of the original plan: orange line; and Y-direction of the measurement dose of the normalized plan with Dolphin: purple line (measurement value: calibration unit (CU))

**Fig. 5:**
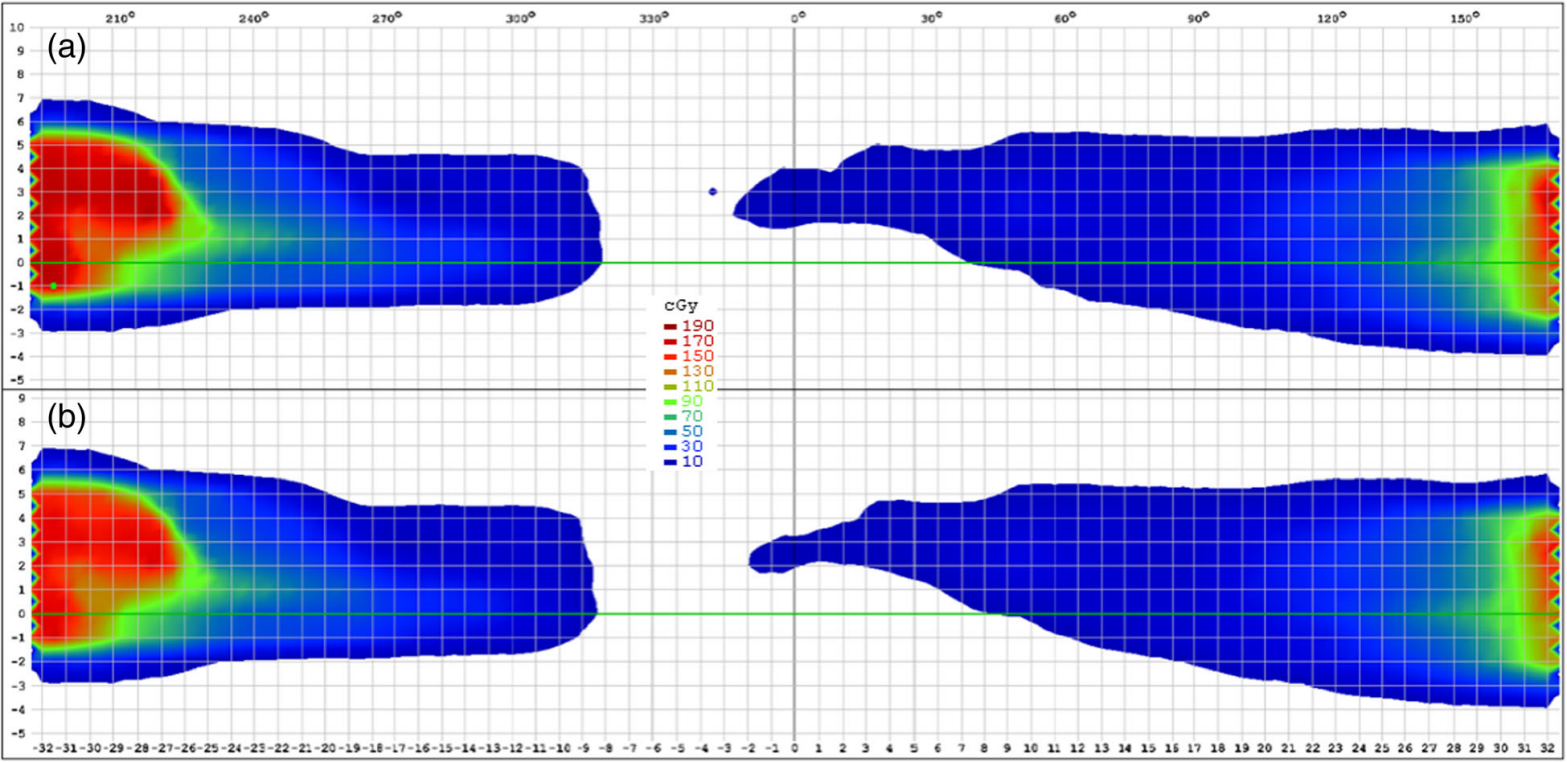
Dose distributions measured by ArcCheck: **a**) original plan without Dolphin and **b**) normalized plan with Dolphin (measurement value: dose (cGy))

**Fig. 6:**
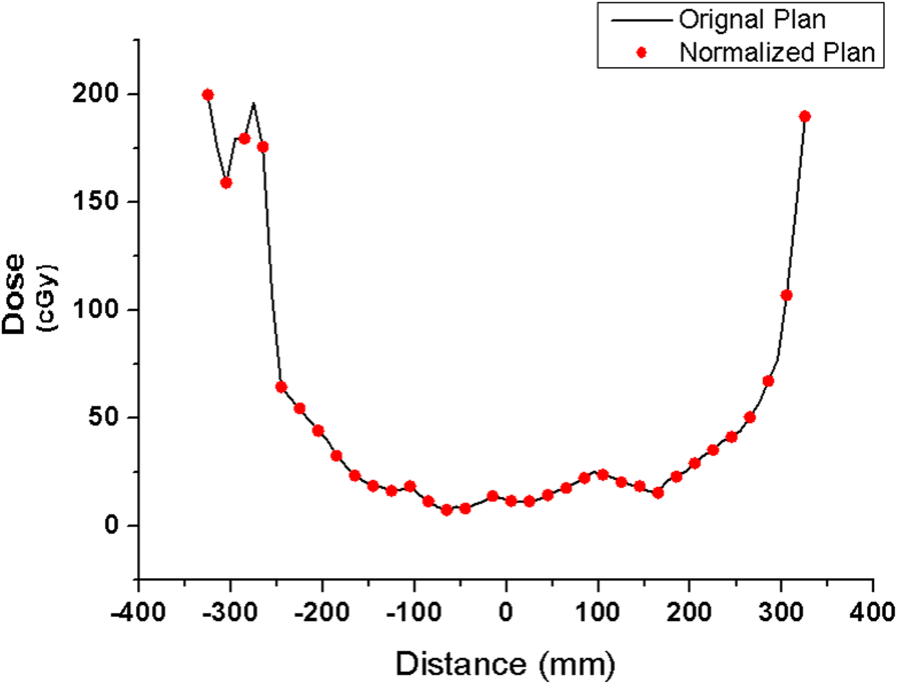
Dose profiles measured by ArcCheck: the red circles and the black line indicate the original and normalized plans, respectively (measurement value: dose (cGy))

Figure 2d shows the measurement results using the new VMAT plan (i.e., normalized by the transmission factor) with Dolphin. The dose distribution was nearly identical to that of the measurement result delivered by the original plan without Dolphin (Fig. 2b). Figure 4 presents the dose profile of the predicted dose of the original plan and the measured portal dosimetry dose of the normalized plan. The dose difference was below 1.8%. Figure 5a shows the dose distribution of the original plan measured by ArcCheck without Dolphin, while Fig. 5b depicts the dose distribution of the normalized plan measured by ArcCheck with Dolphin. Figure 6 shows the measured dose profiles given by ArcCheck.

Table 6 lists the gamma passing rate of the original plan without Dolphin and the normalized plans with Dolphin. The gamma passing rate for all the original and normalized plan measurements obtained through portal dosimetry and with ArcCheck was above 90% with a 2%/2 mm criterion. The minimum gamma passing rate for the original plan verification with the 2%/2 mm criterion was 91.2% with ArcCheck. The maximum was 100% for both portal dosimetry and ArcCheck. Meanwhile, the gamma passing rate for the original plan verification with a 1%/1 mm criterion ranged between 69.1–99.5% and 57.2–98.2% for portal dosimetry and ArcCheck, respectively.

**Table 6:** Gamma passing rates of the transmission factor verification measurements in the clinical plans (unit: %)

Criteria	Original plan without Dolphin Average ± standard deviation (range)		Normalized plan with Dolphin Average ± standard deviation (range)	
	Portal dosimetry	ArcCheck	Portal dosimetry	ArcCheck
2%/2 mm	98.1 ± 2.1 (95.2 ~ 100)	97.3 ± 3.2 (91.2 ~ 100)	97.2 ± 2.8 (94.3 ~ 98.2)	96.9 ± 2.9 (90.1 ~ 98.3)
1%/1 mm	82.9 ± 12.6 (69.1 ~ 99.5)	81.4 ± 15.3 (57.2 ~ 98.2)	79.1 ± 14.8 (73.2 ~ 98.3)	75.8 ± 18.8 (61.9 ~ 94.3)

The minimum gamma passing rate for the ArcCheck measurements for the normalized plan verification with a 2%/2 mm criterion was 90.1%, while the maximum values were 98.2% and 98.3% for portal dosimetry and ArcCheck, respectively. For the SABR plan using portal dosimetry, the gamma passing rate of the original plan without Dolphin was 95.2–97.5% and 69.1–88.1% with 2%/2 mm and 1%/1 mm, respectively. The gamma passing rate of the normalized plan with Dolphin was 94.3–96.1% and 73.2–87.3% with 2%/2 mm and 1%/1 mm, respectively.

## Discussion

In modern radiotherapy, online dosimetry has the potential to improve the accuracy and safety of delivery [15]. Dolphin allows online dosimetry because of the transmission-type detector [11]. Moreover, a 3D dose verification can be performed using Dolphin with COMPASS software [21, 22]. However, Eclipse does not support plan optimization and dose calculation for Dolphin. Therefore, the use of Dolphin in actual treatments is restricted [9].

This study measured the transmission factors of all examined beam energies (6 MV, 6 FFF, 10 MV, and 10 FFF) for various square field sizes. The measured transmission factors varied from 3 × 3 to 20 × 20 cm^2^ for all beam energies. However, in IMRT and VMAT, the change in the field size has been extreme, and small field sizes are generally used for treatment [28]. Therefore, the transmission factors measured for the square field sizes cannot be applied to IMRT, VMAT, or SABR for clinical purposes.

In this work, the MLC transmission factor and the DLG with and without Dolphin were investigated prior to patient-specific QA based on clinical IMRT and VMAT plans. The differences between the values with and with Dolphin were not significant for the MLC transmission factor. For a relative comparison of the QA result, the differences between with and without Dolphin were negligible in the low-dose area. In addition, the DLG was not affected by the detector because the dose profiles were similar in the high-dose gradient region. Such a small difference of the DLG can be neglected [29]. Therefore, the same DLG value was used for the dose calculation.

This study derived the transmission factors provided by Dolphin for actual treatment plans and selected various plans for the measurements. For a relative comparison, the dose distributions were almost identical for measurements with and without the transmission detector. In contrast, an absolute comparison revealed large differences in the dose value because of the attenuation of the detector.

Table 5 shows that the transmission factors were affected by the beam energy only. The delivery technique, MU, and arc range did not influence the transmission factor. Our results showed a maximum difference of 0.8% for the four energies in each transmission factor.

In deriving the transmission factors, the non-negative least-squares problem solver calculated approximately 150,000–850,000 points for each field. The transmission factors of all fields were similar for the same energy. The dose distributions normalized by the transmission factors were also similar to the dose distributions transmitted by Dolphin.

The detector design and the electronic parts inside Dolphin were not uniform [11]. However, each point had similar transmission factors. The maximum dose difference was below 1.7%. Portal dosimetry had a higher resolution than Dolphin because of the detector size of the EPID panel. The interior design of Dolphin did not affect the transmission factors.

The gamma passing rates of the patient-specific verification demonstrated that the plans normalized by the transmission factor can be applied for clinical purposes when a clinically acceptable criterion (i.e., 2%/2 mm) is used [10]. For the SABR plan, the gamma passing rates of the original and normalized plans were poor with the 1%/1 mm criterion because SABR had small fields and high-gradient dose profiles. However, the gamma passing rates of both original and normalized plans were above 90% for all cases when 2%/2 mm criteria were applied. The gamma passing rates of the SABR plans were also within an acceptable range (i.e., above 90%) for clinical purposes.

## Conclusions

This study derived the transmission factor of the Dolphin detector through the measurement of clinical IMRT and VMAT plans. The transmission factors were determined for four energies. However, these factors can vary for nominal energies. This study verified only the transmission factors for the beams of our machine; hence, we recommend the verification of the transmission factors for the beams of each individual machine before clinical use. The Dolphin detector can be an excellent device for online dosimetry if verified transmission factors are used.

## Abbreviations

DD: dose difference
DLG: dosimetric leaf gap
D_max_: depth of maximum dose
DTA: distance to agreement
EPID: electronic portal imaging device
FFF: flattening filter free
IMRT: intensity-modulated radiation therapy
MLC: multileaf collimator
PDD_10_: percentage depth dose at 10 cm depth
PDIP: portal dose image prediction
QA: quality assurance
SABR: stereotactic ablative radiotherapy
TF: transmission factor
TPR 20/10: tissue phantom ratio at the depths of 20 and 10 cm
VMAT: volumetric modulated arc therapy

## References

[1] Georg D, Thwaites D (2017). Medical physics in radiation oncology: new challenges, needs and roles. Radiother Oncol.

[2] Choi CH, Park S-Y, Kim J-i, Kim JH, Kim K, Carlson J (2016). Quality of tri-co-60 MR-IGRT treatment plans in comparison with VMAT treatment plans for spine SABR. Br J Radiol.

[3] Kim J-i, Choi CH, Wu H-G, Kim JH, Kim K, Park JM (2017). Correlation analysis between 2D and quasi-3D gamma evaluations for both intensity-modulated radiation therapy and volumetric modulated arc therapy. Oncotarget.

[4] Vieillevigne L, Molinier J, Brun T, Ferrand R (2015). Gamma index comparison of three VMAT QA systems and evaluation of their sensitivity to delivery errors. Phys Med.

[5] Stevens S, Dvorak P, Spevacek V, Pilarova K, Bray-Parry M, Gesner J (2018). An assessment of a 3D EPID-based dosimetry system using conventional two-and three-dimensional detectors for VMAT. Phys Med.

[6] Liang B, Liu B, Zhou F, F-f Y, Wu Q (2016). Comparisons of volumetric modulated arc therapy (VMAT) quality assurance (QA) systems: sensitivity analysis to machine errors. Radiat Oncol.

[7] Park S-Y, Park JM, Choi CH, Chun M, Han JH, Cho JD (2017). Optimal density assignment to 2D diode array detector for different dose calculation algorithms in patient specific VMAT QA. J Radiat Prot Res.

[8] Crowe S, Kairn T, Middlebrook N, Sutherland B, Hill B, Kenny J (2015). Examination of the properties of IMRT and VMAT beams and evaluation against pre-treatment quality assurance results. Phys Med Biol.

[9] Nakaguchi Y, Ono T, Maruyama M, Shimohigashi Y, Kai Y (2017). Validation of a method for in vivo 3D dose reconstruction in SBRT using a new transmission detector. J Appl Clin Med Phys.

[10] Kim J-i, Choi CH, Park S-Y, An H, Wu H-G, Park JM (2017). Gamma evaluation with portal Dosimetry for volumetric modulated arc therapy and intensity-modulated radiation therapy. Prog Med Phys.

[11] Cheung JP, Perez-Andujar A, Morin O (2017). Characterization of the effect of a new commercial transmission detector on radiation therapy beams. Pract Radiat Oncol.

[12] Ricketts K, Navarro C, Lane K, Blowfield C, Cotten G, Tomala D (2016). Clinical experience and evaluation of patient treatment verification with a transit dosimeter. Int J Radiat Oncol Biol Phys.

[13] van der Bijl E, van Oers RF, Olaciregui-Ruiz I, Mans A (2017). Comparison of gamma-and DVH-based in vivo dosimetric plan evaluation for pelvic VMAT treatments. Radiother Oncol.

[14] Kamerling CP, Fast MF, Ziegenhein P, Menten MJ, Nill S, Oelfke U (2017). Online dose reconstruction for tracked volumetric arc therapy: real-time implementation and offline quality assurance for prostate SBRT. Med Phys.

[15] Thoelking J, Fleckenstein J, Sekar Y, Boggula R, Lohr F, Wenz F (2016). Patient-specific online dose verification based on transmission detector measurements. Radiother Oncol.

[16] Pasler M, Michel K, Marrazzo L, Obenland M, Pallotta S, Björnsgard M (2017). Error detection capability of a novel transmission detector: a validation study for online VMAT monitoring. Phys Med Biol.

[17] Rankine LJ, Mein S, Cai B, Curcuru A, Juang T, Miles D (2017). Three-dimensional dosimetric validation of a magnetic resonance guided intensity modulated radiation therapy system. Int J Radiat Oncol Biol Phys.

[18] McCowan P, Asuni G, van Beek T, van Uytven E, Kujanpaa K, McCurdy B (2017). A model-based 3D patient-specific pre-treatment QA method for VMAT using the EPID. Phys Med Biol.

[19] Miori G, Martignano A, Menegotti L, Valentini A (2016). Evaluation of an integral quality monitor device for monitoring real-time delivery. Phys Med.

[20] Gonod M, Giordan V, ScandiDos, Aubignac L. 6. ScandiDos’s discover system evaluation. Phys Med. 2017;44(S1):30.

[21] Valve A, Keyriläinen J, Kulmala J (2017). Compass model-based quality assurance for stereotactic VMAT treatment plans. Phys Med.

[22] Tomsej M, Monseux A, Baltieri V, Leclercq C, Sottiaux A (2016). Assessment of portal dosimetry accuracy as a QA tool for VMAT clinical treatment plans using dolphin/compass tools. Phys Med.

[23] Miri N, Keller P, Zwan BJ, Greer P (2016). EPID-based dosimetry to verify IMRT planar dose distribution for the aS1200 EPID and FFF beams. J Appl Clin Med Phys.

[24] Yao W, Farr JB (2015). Determining the optimal dosimetric leaf gap setting for rounded leaf-end multileaf collimator systems by simple test fields. J Appl Clin Med Phys.

[25] Middlebrook ND, Sutherland B, Kairn T (2017). Optimization of the dosimetric leaf gap for use in planning VMAT treatments of spine SABR cases. J Appl Clin Med Phys.

[26] Fuangrod T, Rowshanfarzad P, Greer PB, Middleton RH (2015). A cine-EPID based method for jaw detection and quality assurance for tracking jaw in IMRT/VMAT treatments. Phys Med.

[27] Ding L, Deán-Ben XL, Lutzweiler C, Razansky D, Ntziachristos V (2015). Efficient non-negative constrained model-based inversion in optoacoustic tomography. Phys Med Biol.

[28] Oh S, Lewis B, Watson A, Kim S, Kim T (2017). The effect of beam interruption during FFF-VMAT plans for SBRT. Australas Phys Eng Sci Med.

[29] Mullins J, DeBlois F, Syme A (2016). Experimental characterization of the dosimetric leaf gap. Biomed Phys Eng Express.

